# Minimally invasive percutaneous nephrolithotomy improves stone-free rates for impacted proximal ureteral stones: A systematic review and meta-analysis

**DOI:** 10.1371/journal.pone.0171230

**Published:** 2017-02-02

**Authors:** Zi-Ming Gao, Shan Gao, Hong-Chen Qu, Kai Li, Ning Li, Chun-Lai Liu, Xing-Wang Zhu, Yi-Li Liu, Ping Wang, Xiao-Hua Zheng

**Affiliations:** 1 Department of Urological Surgery, Fourth Affiliated Hospital of China Medical University, Shenyang, Liaoning, P.R. China; 2 Department of Gynecology and Obstetrics, Shengjing Hospital of China Medical University, Shenyang, Liaoning, P.R. China; 3 Department of Urological Surgery, Liaoning Cancer Hospital/China Medical University Cancer Hospital, Shenyang, Liaoning, P.R. China; 4 Department of Oncology Surgery, First Affiliated Hospital of China Medical University, Shenyang, Liaoning, P.R. China; 5 Department of Cardre Ward, No. 202 Hospital of People’s Liberation Army, Shenyang, Liaoning, P.R. China; Taipei Medical University, TAIWAN

## Abstract

**Background:**

Urinary stones are common medical disorders and the treatment of impacted proximal ureteral stones (IPUS) is still a challenge for urologists. The aim of this study was to compare the efficacy and safety of minimally invasive percutaneous nephrolithotomy (MI-PCNL) and ureteroscopic lithotripsy (URL) in the treatment of IPUS via a meta-analysis.

**Methods:**

We collected studies using PubMed, Embase, and Cochrane Library from 1978 to November 2016 and analyzed them using Stata 12.0 and RevMan 5.3. Odds ratios (ORs) and standard mean difference (SMD) were calculated for binary and continuous variables respectively, accompanied with 95% confidence intervals (CIs). All study procedures followed the PRISMA guidelines.

**Results:**

Five prospective studies were included in our meta-analysis, with 242 MI-PCNL and 256 URL cases. MI-PCNL was associated with a longer postoperative hospital stay than URL (SMD, 3.14; 95% CI, 1.27 to 5.55). However, no significant difference was observed in operative time (SMD, -0.38; 95% CI, -3.15 to 2.38). In addition, MI-PCNL had higher initial (OR, 11.12; 95% CI, 5.56 to 22.24) and overall stone-free rates (OR, 8.70; 95% CI, 3.23 to 23.45) than URL, along with lower possibilities of surgical conversion (OR, 0.11; 95% CI, 0.03 to 0.49) and postoperative shock wave lithotripsy (OR, 0.06; 95% CI, 0.02 to 0.18). Regarding complications, no significant differences were observed between MI-PCNL and URL (OR, 1.39; 95% CI, 0.93 to 2.10), except for hematuria (OR, 4.80; 95% CI, 1.45 to 15.94).

**Conclusions:**

MI-PCNL is optimal and should be considered as the preferred treatment method for IPUS, as it has better efficacy and a safety profile similar to that of URL. However, further high quality studies with larger sample size are required in future.

## Introduction

Urinary stones are frequently occurring medical disorders worldwide and their incidence has been increasing in recent years.[1] Most of them are upper urinary stones (UUS), including ureteral and renal stones. In the last decade, extracorporeal shock wave lithotripsy (ESWL), ureteroscopic lithotripsy (URL) and percutaneous nephrolithotomy (PCNL) have emerged as common surgical treatment options for UUS, while open surgery is only needed in a few rare circumstances.[2] With advancements in technology, new methods such as, flexible URL, minimally invasive PCNL (MI-PCNL) and laparoscopic ureterolithotomy, have provided more treatment choices to urologists. Within these, MI-PCNL (12-20F) has the advantages of reduced hemorrhage, postoperative pain and hospital stays than traditional PCNL, and has been recommended by many urologists.[3, 4]

Impacted proximal ureteral stones (IPUS) are defined as ureteral stones fixed above the level of fourth lumbar vertebra for at least 1 month.[5] Long-term IPUS may result in hydronephrosis and even renal insufficiency of the affected side. Thus, it is necessary to find a suitable management protocol for relieving obstruction and removing stones simultaneously. ESWL has proven to be less efficient than URL in the treatment of renal and proximal ureteral stones, particularly for critical renal insufficiency.[6–8] With respect to invasiveness, laparoscopic ureterolithotomy still remains as a second-line and remedial measure for other operations.[9] Thus, URL and PCNL are relatively optional methods for the treatment of IPUS. Of which, MI-PCNL might be more suitable for the ureteral stones whose diameter is usually less than 2 cm. In the present study, we aimed to review the comparison in efficacy and safety between URL and MI-PCNL for treatment of IPUS using meta-analysis.

## Materials and methods

### Literature searches and study selection

We searched published articles at PubMed, Embase, and Cochrane Library from 1978 to November 2016. The search terms included “Nephrostomy, Percutaneous OR Minimally Invasive Surgical Procedures OR percutaneous nephrolithot* OR antegrade ureterolithotripsy” and “Ureteroscopy OR ureterolithotripsy OR ureteroscopic lithotripsy OR retrograde ureterolithotripsy” and “Ureteral Calculi OR [impacted (proximal OR upper) ureteral (stone* OR calcul*)]”. Language and sample size were not restricted and the length of follow-up was at least 3 months. In addition, to ensure that we reviewed the literature completely, we tried to find full-text articles of the relevant abstracts and searched for potentially relevant studies from the references of eligible articles. Two reviewers screened the results independently according to the above selection criteria.

### Inclusion and exclusion criteria

The inclusion criteria were as follows: (1) the ureteral stones were diagnosed clearly and impacted at the proximal ureter for at least 1 month; (2) studies that involved the comparison between MI-PCNL and URL; (3) randomized controlled trials (RCTs), cohort studies or other high-quality studies; (4) A follow-up of at least 3 months. Studies were excluded if there were duplications or if they lacked essential data. In addition, letters, comments, reviews, abstracts, or editorial articles were also excluded.

### Data extraction and quality assessment

We extracted the characteristic data from studies using piloted forms, including first author, year of publication, country, median age, sizes of the study population, proportion of male subjects and the stone size. Primary outcomes were initial stone-free rate (SFR; 3–7 days after operation), overall SFR (1–3 months after operation), operation time, and postoperative hospital stay. The secondary outcomes included retreatment and auxiliary procedures (surgical conversion and postoperative ESWL), total cost, and postoperative complications. For analysis of bias risks, Cochrane tools were used for estimating RCT studies, including (1) adequate sequence generation; (2) allocation concealment; (3) blinding; (4) incomplete outcome data addressed; (5) free of selective reporting; (6) free of other bias. The Newcastle-Ottawa Quality Assessment Scale (NOS) was used for cohort or case-control studies, which estimates selection, comparability, and exposure.

### Statistical analysis

We quantitatively compared the primary and secondary outcomes in our meta-analysis. Odds ratios (ORs) with 95% confidence intervals (CIs) were calculated and compared for binary outcomes between MI-PCNL and URL. For continuous variables, results were presented as differences in the mean values between treatments with standard mean difference (SMD) and 95% CIs. Heterogeneity between studies was assessed using the Q test and I^2^; with P<0.1 and I^2^>50%, respectively, considered as significant heterogeneity. Subsequently, the random-effects model was used for analysis. For other analysis, we used the fixed-effects model. If necessary, sensitivity or subgroup analysis was performed to analyze and eliminate the sources of heterogeneity. Publication bias was assessed via construction of a funnel plot of operative time, postoperative hospital stay, overall SFR, and total complications. All the above analyses were performed using Stata 12.0 and RevMan 5.3. The P value was calculated as two-sided, and P<0.05 was considered statistically significant.

## Results

**Fig 1:**
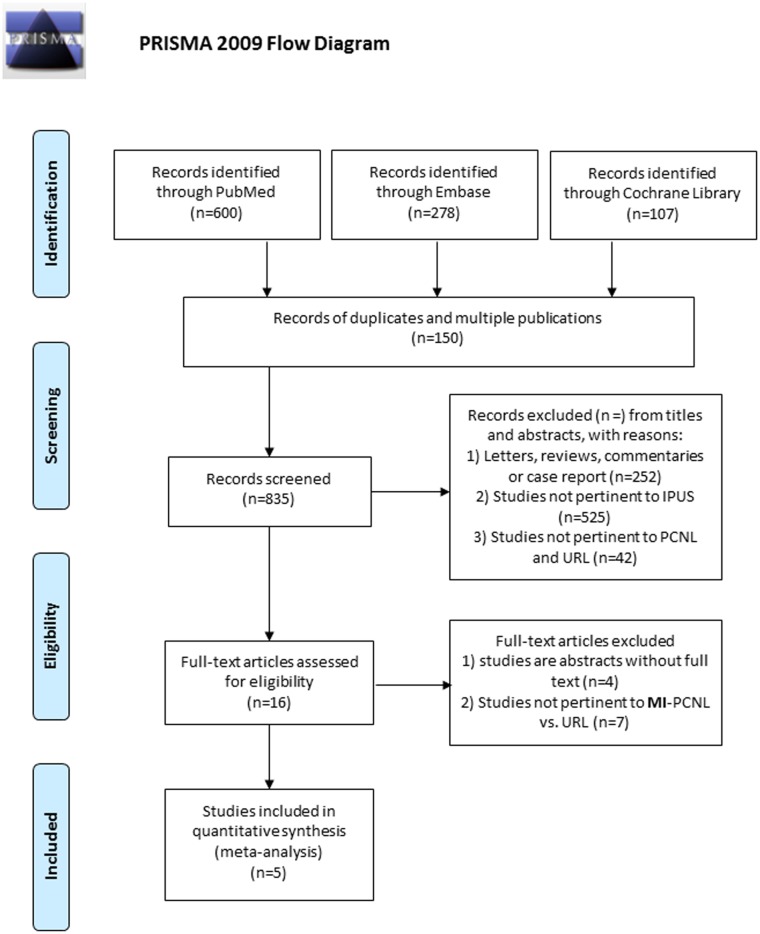
The flowchart showing study search and selection process.

**Fig 2:**
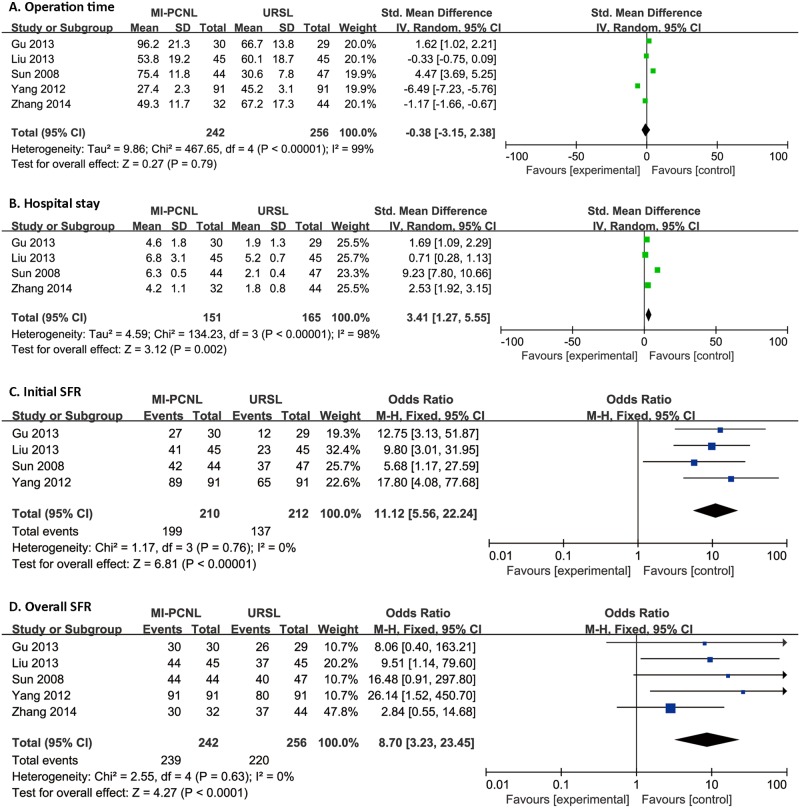
Forest plot of primary outcomes between MI-PCNL and URL. Operative time (A), hospital time (B), initial SFR (C), and overall SFR (D).

**Fig 3:**
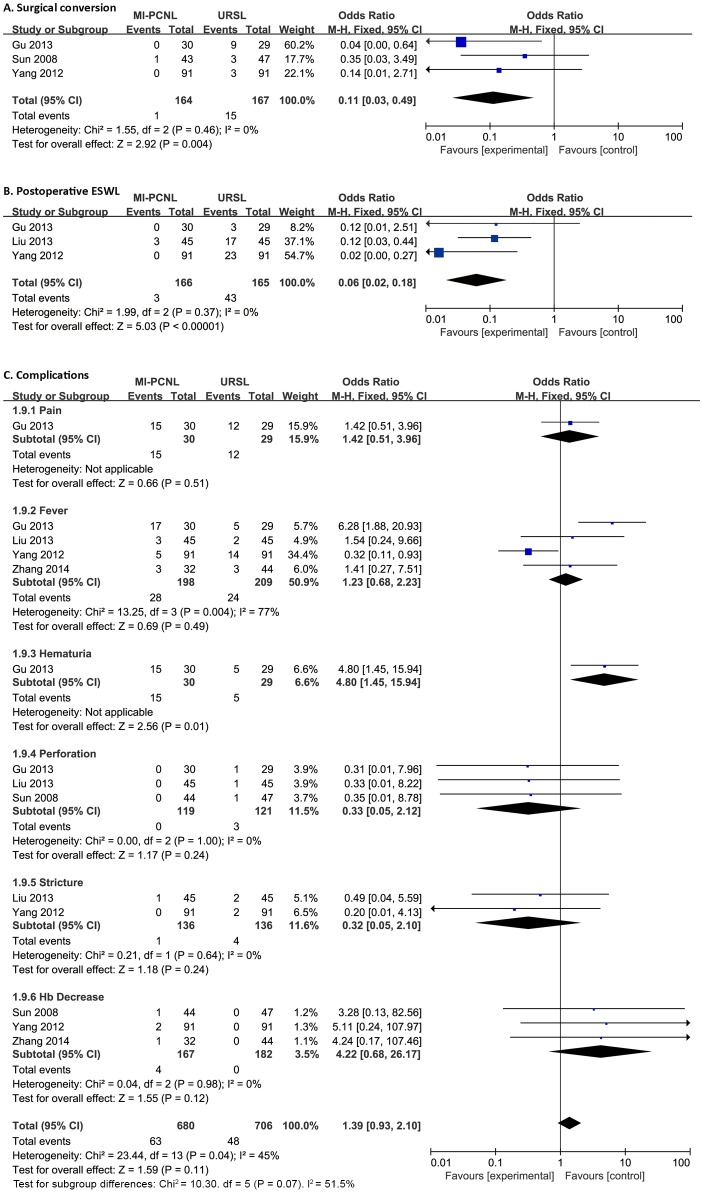
Forest plot of secondary outcomes between MI-PCNL and URL. Surgical conversion (A), postoperative ESWL (B), and complications (C).

**Fig 4:**
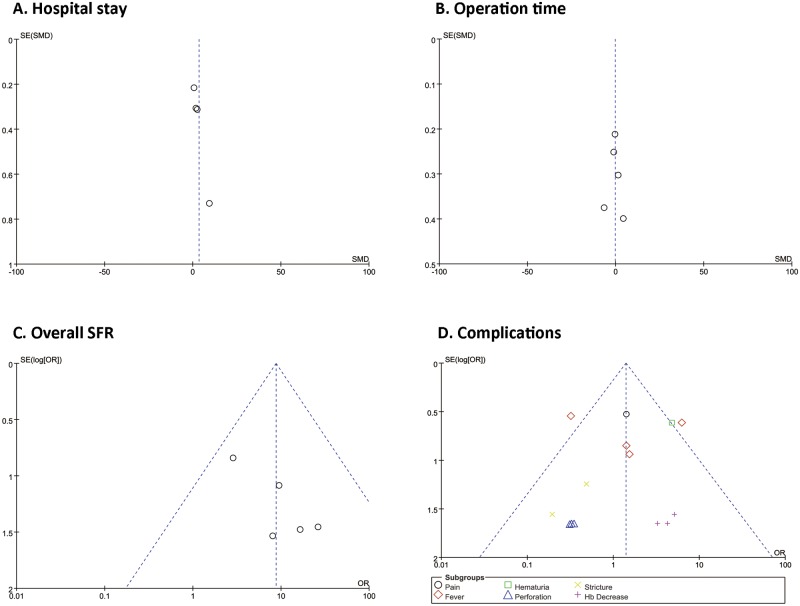
Funnel plot evaluating the publication bias of studies. Hospital Stay (A), operation time (B), overall SFR (C), and complications (D).

All procedures followed the PRISMA guidelines (S1 Table); the search and selection process is presented in Fig 1. A total of four RCTs and one non-randomized concurrent controlled trial (Non-R) studies were included in our meta-analysis, with 242 MI-PCNL and 256 URL cases.[5, 10–13] In the URL group, 183 patients underwent surgery with a rigid ureteroscope (R-URL), while the other 73 patients received treatment with the flexible ureteroscope (F-URL). The original characteristics and data from the selected publications are listed in Table 1, and the quality evaluation is presented in the supporting information. Forest plots of the meta-analysis on efficacy and safety are presented in Figs 2–4. In addition, our analysis had no significant publication bias.

**Table 1:** Original characteristics and data of included articles.

Author	Year	Country	Type	Surgery	Size of fistulas	Number	Male/Female	Age	Stone Size (mm/mm^3^)
Sun	2008	China	RCT	MI-PCNL	14-16F	44	30/14	40.4±8.4	14.7±2.0
				R-URL		47	31/16	39.6±7.3	14.6±1.8
Yang	2012	China	RCT	MI-PCNL	16F	91	53/38	45.2±14.7	158.7±96.8
				R-URL		91	54/37	46.4±15.1	134.2±83.3
Gu	2013	China	RCT	MI-PCNL	12-18F	30	17/13	42.5±10.1	16.23±2.5
				F-URL		29	11/18	44.22±13.0	17.27±2.5
Liu	2013	China	RCT	MI-PCNL	None	45	23/22	46.35±10.3	146.85±30.3
				R-URL		45	25/20	43.41±10.1	148.13±27.5
Zhang	2014	China	Non-R	MI-PCNL	18-20F	32	24/8	42.7±13.6	15.6±2.5
				F-URL		44	29/15	43.3±11.0	14.9±2.3

### Primary outcomes

#### Operation time

All five studies were included in the forest plot (Fig 2A). Heterogeneity analysis showed I^2^ = 99% and P = 0.000<0.05. Random-effect meta-analysis was applied because of the severe heterogeneity. There was no significant difference in operation time between the two groups (SMD, -0.38; 95% CI, -3.15 to 2.38).

#### Postoperative hospital stay

Four studies were included in the forest plot (Fig 2B). The results of heterogeneity analysis were I^2^ = 98% and P = 0.000<0.05, which was severe; thus, random-effect analysis was applied, which showed that MI-PCNL may be associated with a longer postoperative hospital stay than URL (SMD, 3.14; 95% CI, 1.27 to 5.55).

#### SFR

Four studies were included in the forest plot of initial SFR (Fig 2C). Heterogeneity analysis showed I^2^ = 0.0%, and P = 0.76>0.05. The fixed-effects model of meta-analysis was performed, which showed that MI-PCNL has a higher initial SFR compared to URL (OR, 11.12; 95% CI, 5.56 to 22.24). In terms of overall SFR, all five studies were included in the forest plot (Fig 2D). Heterogeneity analysis showed I^2^ = 0.0% and P = 0.76>0.05. There was no heterogeneity, and the fixed-effects model showed that MI-PCNL also has higher overall SFR compared to URL (OR, 8.70; 95% CI, 3.23 to 23.45).

### Secondary outcomes

#### Retreatment and auxiliary procedures

Three studies were included in the forest plot of surgical conversion (Fig 3A). Heterogeneity analysis revealed I^2^ = 0.0% and P = 0.458>0.05. No heterogeneity existed and the fixed-effect model of meta-analysis was performed, which showed that MI-PCNL has a lower risk of surgical conversion compared to URL (OR, 0.11; 95% CI, 0.03 to 0.49). In addition, three studies were included in the forest plot of postoperative ESWL (Fig 3B). There was also no heterogeneity between these studies (I^2^ = 0.0%, P = 0.37>0.05), and the fixed-effect model showed that postoperative ESWL was more common in URL compared to MI-PCNL (OR, 0.06; 95% CI, 0.02 to 0.18).

#### Cost

Apart from efficacy and safety, cost is an important concern for patients. In our analysis, only one study reported the cost comparison between MI-PCNL and URL, which revealed that the cost of MI-PCNL was higher than URL (SMD, 3.69; 95% CI, 3.21 to 4.17). [11]

#### Postoperative complications

Grade IV Clavien system complications were rare in both methods. The comparison on pain (OR, 1.42; 95% CI, 0.51 to 3.96), fever (I^2^ = 77%, P = 0.004; OR, 1.23; 95% CI, 0.68 to 2.23), hematuria (OR, 4.80; 95% CI, 1.45 to 15.94), perforation (I^2^ = 0.0%, P = 1.00; OR, 0.33; 95% CI, 0.05 to 2.12), stricture (I^2^ = 0.0%, P = 0.64; OR, 0.32; 95% CI, 0.05 to 2.10), and hemoglobin decrease (I^2^ = 0.0%, P = 0.98; OR, 4.22; 95% CI, 0.68 to 26.17) were analyzed in our forest plot (Fig 3C), which showed that there were no significant differences between MI-PCNL and URL, except for hematuria. In addition, the total incidence rates of complications in MI-PCNL and URL were also similar (I^2^ = 45%, P = 0.04; OR, 1.39; 95% CI, 0.93 to 2.10).

### Risk of bias

A quality review of included studies was performed by Cochrane tools or NOS, and most studies had a low risk of bias (S1 Fig, S2 Table). For all four RCT studies, no bias but blinding was significant. The NOS score of the cohort study was eight, which suggested that it described high-quality research.

### Publication bias

Analysis of publication bias was performed with a funnel plot of part outcomes, which showed that no publication bias existed in our analysis (Fig 4). However, considering the small number of included studies, we could not ensure its accuracy.

## Discussion

Recently, the incidence of urinary stones has increased significantly due to changes in people’s diets and lifestyle. Among different urinary stones, IPUS is a special type which indicates long-term retention of stones at the proximal ureter. Urination is always difficult for IPUS patients and if not treated in time, renal failure and other life-threatening diseases might occur. Ureteral polyp is the major cause of IPUS and it always needs to be treated at the same time. Recently, PCNL and URL have emerged as the two common methods used for treatment of IPUS. However, there are advantages and disadvantages of both procedures, and which procedure is optimal for IPUS remains unclear and challenging for urologists.

URL could be performed as a routine method through natural tracts for treating distal ureteral and bilateral stones effectively. In addition, the application of F-URL could reduce invasiveness of the ureteroscope to some extent and has been used for small renal stones.[14] However, URL has its natural defects. When treated by URL, stones, especially the proximal ones, can easily move back to the renal pelvis. For example, Chow et al reported that about 25% patients who underwent URL suffered from complications of stone migration. Moreover, if the patients’ ureters are constrictive or anfractuous, it is difficult for the ureteroscope to insert and reach stones. In recent years, the technology of PCNL has matured and can avoid these disadvantages of URL. In addition, urologists can use most lithotripters through the PCN tunnel and even treat the associated renal stones simultaneously. However, postoperative pain and hemorrhage are still common complications for all types of PCNL. Thus, MI-PCNL with a smaller size of percutaneous tract and less trauma, has already been popular in treating renal and proximal ureteral stones.

Most conclusions of our analysis were similar to other empirical literature.[15–17] In our study, no significant difference was observed in operative time, but MI-PCNL might require longer postoperative hospital stays than URL. In terms of efficacy, MI-PCNL had higher initial and overall SFRs, along with lower risk of surgical conversion and postoperative SWL than URL, which reflected the higher operative success rate of MI-PCNL. With respect to safety, no significant differences were observed in the comparison of total complications between the two methods. Essentially, only hematuria was more common in MI-PCNL than URL. Notably, perforation, stricture and infectious shock only existed in the URL group. Within all five studies, there were three patients with perforation, four with stricture and one with infectious shock among the 256 URL cases. In addition, severe hemorrhage, which is a common complication of PCNL, only occurred in the MI-PCNL group. A total of three MI-PCNL cases needed blood transfusion, and one of them underwent arterial embolization. Thus, we propose that higher Clavien grade complications may be more common in URL than in MI-PCNL, and urologists should always avoid these complications despite improvements in technology. Moreover, Mehmet et al have reported that kidney damage from PCNL tracts was negligible using radionuclide renography.[18] In conclusion, we present that MI-PCNL is more effective and safe for treating IPUS than URL (irrespective of R-URL or F-URL), and should be the preferred treatment method.

To minimize the occurrences of part complications, we also searched related literature. Liu et al suggested that tubeless PCNL could reduce postoperative pain significantly.[19] With respect to fever, perioperative administration of anti-inflammatory drugs is necessary to reduce the risk of infection and infective shock. Furthermore, Hamamoto et al. reported that a combined surgery with MI-PCNL and URL was better than monotherapy, which is worth paying attention to.[15]

Heterogeneity is an important component of meta-analysis. In the primary outcomes of this study, high heterogeneity (I^2^>75) was present only in the comparison of operative time and hospital stay. Differences in surgical skills and treatment concepts were the main reasons for heterogeneity. Undeniably, operative levels remain uneven in different regions of the world because of economic factors, which would directly affect operative time. In addition, clinicians have different standards in the treatment of diseases, with certain urologists prolonging postoperative hospital stay to ensure that the patients are well before they leave. However, others advocate day-operation, leaving patients with shorter hospital stay. In addition, the heterogeneity of postoperative fever analysis was high, but considering the few included studies, we did not perform sensitivity or subgroup analysis.

Our study is the first meta-analysis to compare MI-PCNL and URL for treating IPUS. MI-PCNL is an emerging urological technology that is rarely used for the treatment of IPUS at present. Thus, the results of our study could provide a new, effective, and safe choice for surgeons in the treatment of IPUS. In addition, we only included five prospective studies with low heterogeneity, which ensure the reliability of our results.

However, our study has a few limitations. First, the small number of studies included might reduce the persuasiveness of conclusions. For example, only two or three studies were included in the comparison of pain, stricture, and blood transfusion. Therefore, it is worth further examining these parameters using large cohorts and studies. Second, almost all the studies that compared the surgical methods did not have a double-blind design because of clinical requirement, which reduces the quality of studies included. Third, all populations in our analysis were Chinese, which limits the wide applicability of our results.

## Conclusions

MI-PCNL has better efficacy and similar safety compared to URL. Thus, MI-PCNL is the optimal method among these two for treatment of IPUS. However, studies with larger sample size and detailed records are needed to further validate our results.

## Supporting information

S1 Fig Quality review of Cochrane tools. (TIF)

S1 Table PRISMA 2009 Checklist. (DOC)

S2 Table Quality review of NOS. (XLSX)

S3 Table Search strategies. (DOCX)

S4 Table The data of all studies. (XLS)
