# Supplementary figures and images for: Minimally invasive percutaneous nephrolithotomy improves stone-free rates for impacted proximal ureteral stones: A systematic review and meta-analysis
Source: PLoS One. 2017 Feb 2;12(2):e0171230. doi: 10.1371/journal.pone.0171230 (PMC5289591; doi:10.1371/journal.pone.0171230)

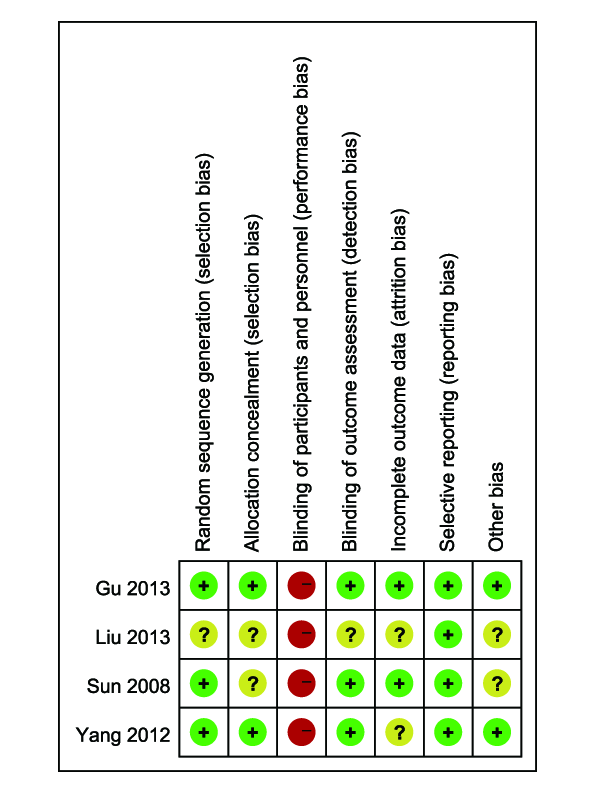

Supplement: S1 Fig — (TIF) [file pone.0171230.s001.tif]
