# Supplementary material for: Minimally invasive percutaneous nephrolithotomy improves stone-free rates for impacted proximal ureteral stones: A systematic review and meta-analysis
Source: PLoS One. 2017 Feb 2;12(2):e0171230. doi: 10.1371/journal.pone.0171230 (PMC5289591; doi:10.1371/journal.pone.0171230)

**miPCNL vs. URL Search strategies**

**1. PubMed**

#1 Nephrostomy, Percutaneous [MeSH Major Topic]

#2 Minimally Invasive Surgical Procedures [MeSH Major Topic]

#3 (percutaneous) AND ((nephrolithot*) OR antegrade ureterolithotripsy)

#4 (#1 or #2 or #3)

#5 Ureteroscopy [MeSH Major Topic]

#6 ((ureterolithotripsy) OR ureteroscopic lithotripsy) OR retrograde ureterolithotripsy

#7 (#5 or #6)

#8 Ureteral Calculi [MeSH Major Topic]

#9 (((impacted) AND ((proximal) OR upper)) AND ureteral) AND ((stone*) OR calcul*)

#10 (#8 or #9)

#12 (#4 and #7 and #10)

Search (((((Nephrostomy, Percutaneous [MeSH Major Topic]) OR Minimally Invasive Surgical Procedures [MeSH Major Topic]) OR (((percutaneous) AND ((nephrolithot*) OR antegrade ureterolithotripsy))))) AND ((Ureteroscopy [MeSH Major Topic]) OR ((((ureterolithotripsy) OR ureteroscopic lithotripsy) OR retrograde ureterolithotripsy)))) AND ((Ureteral Calculi [MeSH Major Topic]) OR (((((impacted) AND ((proximal) OR upper)) AND ureteral) AND ((stone*) OR calcul*))))

Total 600 papers in PubMed


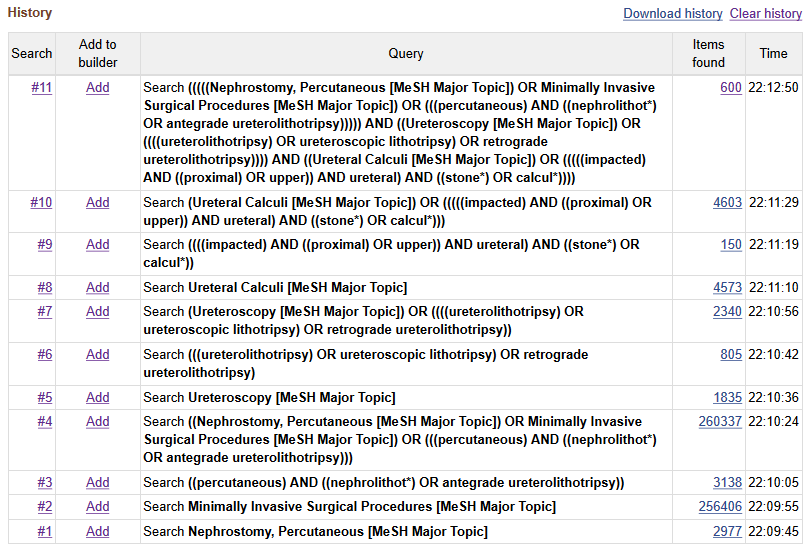


**2. Cochrane Library**

#1 MeSH descriptor: [Nephrostomy, Percutaneous] explode all trees

#2 MeSH descriptor: [Minimally Invasive Surgical Procedures] explode all trees

#3 (percutaneous) AND ((nephrolithot*) OR antegrade ureterolithotripsy)

#4 (#1 or #2 or #3)

#5 MeSH descriptor: [Ureteroscopy] explode all trees

#6 ((ureterolithotripsy) OR ureteroscopic lithotripsy) OR retrograde ureterolithotripsy

#7 (#5 or #6)

#8 MeSH descriptor: [Ureteral Calculi] explode all trees

#9 (((impacted) AND ((proximal) OR upper)) AND ureteral) AND ((stone*) OR calcul*)

#10 (#8 or #9)

#11 (#4 and #7 and #10)

Total 107 papers in Cochrane Library


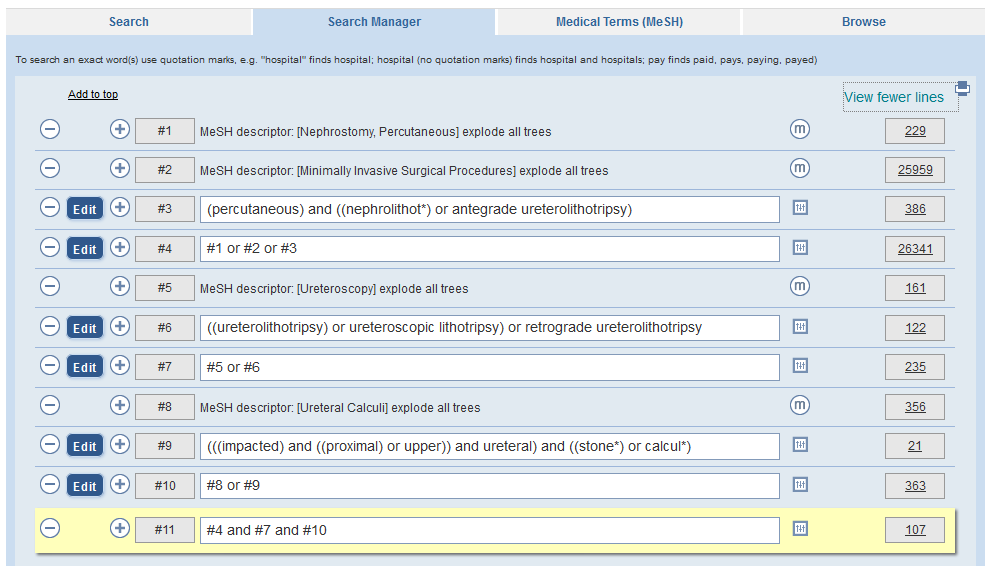


**3. Embase**

1 'percutaneous nephrolithotomy'/exp or 'minimally invasive surgery'/exp

2 (percutaneous) AND ((nephrolithot*) OR antegrade ureterolithotripsy)

3 1 or 2

4 'ureteroscopy'/exp

5 ((ureterolithotripsy) OR ureteroscopic lithotripsy) OR retrograde ureterolithotripsy

6 4 or 5

7 'ureter stone'/exp

8 impacted and (proximal or upper) and ureteral and (stone* or calcul*)

9 7 or 8

10 3 and 6 and 9

Total 278 papers in Embase


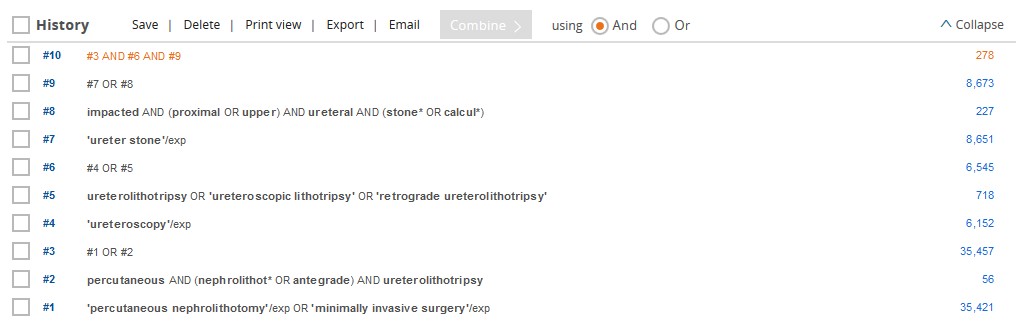

Supplement: S3 Table — (DOCX) [file pone.0171230.s004.docx]
